# ChatGPT, the future of healthcare research writing: Is it a double-edged sword?

**DOI:** 10.34172/joddd.2023.37147

**Published:** 2023-07-17

**Authors:** Abhishek Lal

**Affiliations:** Department of Medicine, The Aga Khan University, Karachi, Pakistan

## To Editor,

 Technological advancement has been on the rise aiming to improve scientific research. In less than 2 months of its official launch, ChatGPT was taken every field by storm, including research. ChatGPT is an artificial intelligence (AI) based technology developed on OpenAI that gathers information from all over the internet, including conversation, by humans and delivers outcomes as per the user’s query. According to the query of the user, ChatGPT gathers information from every possible source and presents that combination of words in a meaningful way. Ever since ChatGPT has been released to the public, it has attracted numerous investors, researchers, and people from all sorts of backgrounds to explore the tremendous and powerful AI-based technology.

 Since its release, ChatGPT has been tested by various experts to evaluate how meaningful sentences can be extracted when a certain query is searched upon. It is certain that the introduction of ChatGPT will have a huge impact on researchers and how research is conducted, entering a new era. ChatGPT has been subjected to some excitement and controversy at the same time.

 ChatGPT can help in assisting the researchers in the literature review, analysis of data, and generation of hypotheses as well. Several advantages are associated with ChatGPT such as the analysis of large quantities of data that includes medical reports, research articles, along with the records of the patients as well.^1^ The output offered by ChatGPT offers new insights that can help in writing medical research such as causes, symptoms, and treatment options of numerous pathologies. When writing a research paper, ChatGPT supposedly is able to extract specific and relevant information and presenting in an ordered and meaningful manner. The search on ChatGPT creates pathways for new topics of research and hypothesis that can help in working on unexplored research areas. By using ChatGPT, various gaps in the field of healthcare can be filled by generation and exploring new hypotheses and ideas. Furthermore, ChatGPT can be helpful for the creation of clinical decision support systems, where the pattern of records of patients is analysed and possibly enhancing the health care of the patient.

**Figure 1:**
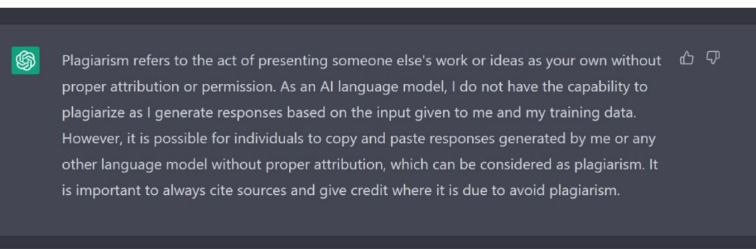
Plagiarism in ChatGPT.

 Despite the advantages associated with ChatGPT, not all researchers agree with its use and introduction in healthcare research. One of the potential problems associated with ChatGPT is plagiarism. When asked by ChatGPT about plagiarism, although it says that it does not have the capacity to plagiarize, but the text should be cited in order to avoid plagiarism, as shown in Figure 1.

 The current conventionally used plagiarism detectors might not the equipped to detect plagiarism associated with ChatGPT. Another problem associated with the use of ChatGPT by a researcher is the generation of text quite similar to the manuscripts that have already been published. Such detrimental effects can tamper with the results of published studies which may mislead readers as well as researchers. Secondly, the text generated can have a misleading context as compared to the query entered by the user since ChatGPT analyses a large amount of data, not a specific topic. In a recent preprint analyzing abstracts written by ChatGPT to original abstracts, about 32% of the blinded reviewers were misled by it.^2^

 Critical thinking has been one of the most important pillars in terms of scientific writing in terms of healthcare research. Since the introduction of ChatGPT, there can be a reduction in critical thinking by researchers as the AI-based technology offers quick solutions. This can have a serious impact on the novelty and innovations of the researchers.

 When used sensibly, ChatGPT has many advantages associated with it as mentioned in this paper. However, there are many pitfalls associated with it so it should be used with caution at the moment. In terms of research, we as healthcare researchers need to explore ways where such AI-based technology can be successfully integrated into the research work without jeopardizing the scientific basis of the work.

## Competing Interests

 None to declare.

## Ethical Approval

 Not applicable.

## Funding

 None.

## References

[1] Kung TH, Cheatham M, Medenilla A, Sillos C, De Leon L, Elepaño C (2023). Performance of ChatGPT on USMLE: potential for AI-assisted medical education using large language models. PLOS Digit Health.

[2] Gao CA, Howard FM, Markov NS, Dyer EC, Ramesh S, Luo Y, et al. Comparing scientific abstracts generated by ChatGPT to original abstracts using an artificial intelligence output detector, plagiarism detector, and blinded human reviewers. bioRxiv [Preprint]. December 27, 2022. Available from: https://www.biorxiv.org/content/10.1101/2022.12.23.521610v1.

